# Strategies Developed to Induce, Direct, and Potentiate Bone Healing

**DOI:** 10.3389/fphys.2017.00927

**Published:** 2017-11-14

**Authors:** Anne-Margaux Collignon, Julie Lesieur, Christian Vacher, Catherine Chaussain, Gael Y. Rochefort

**Affiliations:** ^1^EA 2496 Orofacial Pathologies, Imaging and Biotherapies, Dental School Faculty, Life Imaging Platform (PIV), University Paris Descartes, Montrouge, France; ^2^Department of Odontology, University Hospitals PNVS, Assistance Publique Hopitaux De Paris, Paris, France; ^3^Department of Maxillofacial Surgery, Beaujon Hospital, Assistance Publique Hopitaux De Paris, Paris, France

**Keywords:** bone healing, scaffold, stem cells, biomolecules, bone regeneration

## Abstract

Bone exhibits a great ability for endogenous self-healing. Nevertheless, impaired bone regeneration and healing is on the rise due to population aging, increasing incidence of bone trauma and the clinical need for the development of alternative options to autologous bone grafts. Current strategies, including several biomolecules, cellular therapies, biomaterials, and different permutations of these, are now developed to facilitate the vascularization and the engraftment of the constructs, to recreate ultimately a bone tissue with the same properties and characteristics of the native bone. In this review, we browse the existing strategies that are currently developed, using biomolecules, cells and biomaterials, to induce, direct and potentiate bone healing after injury and further discuss the biological processes associated with this repair.

## Introduction

The vertebrate skeleton is constituted by stiff bone organs with osseous tissue, bone marrow, endosteum, periosteum, cartilage, nerves, and vascular channels. Basically, bone tissue can be separated into an inorganic part (60–70%), water (5–10%) and an organic matrix of cells, collagen, and other matrix associated proteins (the remaining portion). These composition and configuration may greatly vary depending on the anatomical location, supported load, age, gender, and the possible disease situation (Boskey et al., 2016). The mineral phase of bone is made of inorganic mineral salts and ions: calcium, phosphate, carbonate, citrate, hydroxyl, and other ions (magnesium, sodium, and fluoride), organized into hydroxyapatite nanocrystals, with a length of 25–50 nm (Glimcher, 1987). The organic component is composed of more than 90% of collagen type-1 and several non-collagenous proteins including growth factors, cytokines, osteocalcin, osteonectin, osteopontin, phosphoproteins, proteoglycanes, bone morphogenic proteins, and phospholipids (Boskey and Posner, 1984; Boskey, 2013).

In addition, bone contains several cellular elements: pre-osteoblasts, osteoblasts, osteocytes, and osteoclasts. Deriving from multipotent stromal mesenchymal (stem) cells, osteoblasts are mononucleated, with a cuboidal shape. Localized at the surface of bone tissue, these cells are responsible for bone formation by producing the organic bone matrix (un-mineralized osteoid matrix) and directing the initiation of the mineralization process through the secretion of enzymes such as the alkaline phosphatase. During these secretion and mineralization processes, around 10–20% of the osteoblasts remain alive and become embedded into the matrix that they have secreted, and mature into osteocytes (Rochefort et al., 2010). Compared to osteoblasts, osteocytes are smaller, with a higher nucleus to cytoplasm ratio and exhibit several dentritic processes that form the lacuna-canalicular system allowing intercellular communication (Knothe Tate et al., 2004; Rochefort, 2014). As mechanosensitive cells osteocytes are able to sense mechanical variations within the bone tissue and to send regulatory signals that will initiate and regulate the directed bone remodeling (Lanyon, 1993; Rochefort and Benhamou, 2013; Bonewald, 2017). The process of bone resorption is supported by osteoclasts that secrete H^+^ and enzymes at the resorption site to reduce the pH allowing enzymes to cleave of the bone matrix. Deriving from bone marrow monocyte precursors, osteoclasts are large, multinucleated, polarized cells with phagocytic properties (Ikeda and Takeshita, 2016).

Osseous tissue may be structurally organized into trabecular (cancellous) bone or cortical (lamellar) bone (Nishiyama and Boyd, 2011). Trabecular bone, found at the metaphysis and the epiphysis of long bones and at the interior of cuboid bones, is a network of interconnected of bone segments with plate and rod configuration, ranging between 50 and 90% of “empty” space that is filled with bone marrow tissue. Cortical tissue, forming a compact and homogeneous macrostructure, is located at the diaphysis of long bones and at the bone surface with a thickness varying according to the bone anatomical location. Structurally and functionally, cortical bone is composed of individual units named osteons or Haversian systems that are organized along the bone. Osteons are centered on central channels with vessels and nerves providing nutrients and oxygen to the cells.

While the human population is aging, the incidence of bone trauma will inevitably increase as well. Susceptibility to bone fracture is also majored by the increasing number of women and men with osteoporosis. In the US, about 5 and 10% of bone fractures exhibit a disunion or late healing and therefore remain a key management in orthopedic surgery (Einhorn, 1995). The current surgical method for large bony defect reconstruction implies the harvesting of autologous bone segments (e.g., radius, fibula, iliac crest, scapula…), leading to extended hospitalization, associated morbidity and complications, and increasing direct and indirect healthcare costs. Thus, there is an important clinical need for the elaboration of new healing possibilities for bone prevention and repair of bone fractures (Knothe Tate et al., 2011, 2013). As an alternative to autologous bone graft, recent developments are currently examined to locally induce and stimulate the bone healing process, but also to fill the bone defect with allogenic materials (bone material sourced from a donor) or by tissue engineering biomaterials, mimicking the local microenvironment in order to stimulate the physiological bone development, without donor site morbidity (Veronesi et al., 2015; Russo et al., 2017).

The objective of this review is to describe and discuss the current strategies developed to potentiate healing bone processes after injuries, using biomolecules, cells, and biomaterials.

### Bone regenerative strategies: biomolecules, cells, and biomaterials

When injured, bones have the rare property of endogenous self-repair by regenerating new bone without forming a fibrotic scar that would modify their mechanical characteristics (Kalfas, 2001). Consequently, the healing process of adult bones follows the whole steps of the bone formation during embryogenesis and organogenesis, where the freshly renewed bone is finally not distinguishable from the initial tissue. However, the improvement of this healing procedure is mandatory to guarantee the fast and suitable restoration of bone properties and functions in several pathological conditions such as inadequate immobilization, impaired blood supply, periosteum excessive damage, infection, mineral and vitamin lacks, primary pathologies, specific medications, or radiation (Chimutengwende-Gordon and Khan, 2012; Shekkeris et al., 2012). Beside standard therapies that include mechanical support (e.g., casts, nails, plates, and screws) to treat bone fractures/defects, other approaches currently developed and used to direct and further enhance bone restoration are primarily centered on the utilization of: (1) active elements or biomolecules, (2) cell-based treatments, and (3) biomaterials.

### Biomolecules and biotherapies

Biomolecules used in bone regenerative therapies are mostly growth factors, cytokines, or hormones (Vo et al., 2012; Katagiri et al., 2013). There are acting as biochemical signals triggering cellular functions, such as migration, proliferation, differentiation, secretion, or apoptosis, among others.

The most studied osteogenic factors are members of the transforming growth factor-β (TGF-β) superfamily, and principally bone morphogenetic proteins (BMPs) (Lissenberg-Thunnissen et al., 2011; Carreira et al., 2015). These cytokines are acting on skeletal tissue formation and growth, but also in adulthood during bone healing process, by promoting the osteoblastic differentiation of mesenchymal stem cells, stimulating the chondrocyte and osteoblast proliferation, and increasing the production of extracellular matrix. In the skeleton, BMPs are located among the collagen fibers within the bone matrix, in the periosteum and in the bone marrow. After a fracture, BMPs are released from the bone matrix and diffuse to induce and stimulate osteoprogenitors that, in turn, will produce more BMPs. BMP-2, -4 and -7 are thus able to stimulate bone formation *in vitro* and *in vivo* at heterotopic sites, while BMP-1, -2 and -3 can increase the *in vitro* production of collagen type I and osteocalcin from osteoblastics cells and induce the development of mineralized bone nodules. Because of their great therapeutic possibilities, BMPs were largely used alone or in combination with porous scaffolds to promote healing and growth in the typical management of bone disunions, open fractures, spinal fusions, and maxillofacial damages (Kang et al., 2012; Rahman et al., 2014; Wang et al., 2014). However, heterotopic bone formation, osteolysis, radiculitis, and retrograde ejaculation were also reported when using BMPs (Tannoury and An, 2014; Kang et al., 2017). TGFs-β are able to promote the chondrocyte and osteoblast proliferation and also differentiation of mesenchymal cells into chondrocytes. These factors are of crucial importance in coupling bone formation and resorption processes by increasing bone resorption at higher concentrations.

Other factors, FGFs, IGFs, PDGFs, and VEGF, are extensively tested for their osteogenic and angiogenic properties in bone repair (Wildemann et al., 2007). FGFs can stimulate the mesenchymal cell, osteoblast, and chondrocyte proliferation, but also enhance tissue growth due to their angiogenic properties. Within the FGF family, FGF-2 or bFGF is the most studied cytokine regarding bone healing applications (Bai et al., 2013). IGFs are able to promote the proliferation and the collagen matrix synthesis and secretion from osteoblast and chondrocytes (Bai et al., 2013; Moller et al., 2013). Depending on their concentrations, PDGFs are chemotactic and mitogenic factor that can either promote chondrocyte and osteoblast proliferation, or induce bone resorption (Bai et al., 2013).

In addition, several factors, including the parathyroid hormone, the growth hormone, steroids, the calcitonin or the vitamin D, are also used, alone or associated with other elements, in systemic applications to stimulate osteogenesis, angiogenesis, and osteoblast differentiation during bone healing (Ellegaard et al., 2010; Suchak and Soory, 2013; Prideaux et al., 2015; Verdonk et al., 2015).

It has also been recently reported that osteocytes are producing sclerostin, an antagonism of the Wnt signaling pathway, which constitutively restricts the bone formation. Several studies thus demonstrated that sclerostin inhibition using anti-sclerostin monoclonal antibodies can increase the bone formation of all bone compartments, including the trabecular, endosteal, intracortical, and periosteal faces. These sclerostin antibodies can thus promote a bone mass increase and bone strength improvement, as well as enhancing fracture consolidation and healing of both non-critical and critical size skeletal defects in numerous studies in animal models (Jawad et al., 2013; Virk et al., 2013; Alaee et al., 2014; Tinsley et al., 2015), but also in human studies (Padhi et al., 2011; Costa et al., 2014; McColm et al., 2014; Recker et al., 2015).

At last, microRNAs (miRNAs) have been recently reported as regulators of skeleton growth [15, 31–34] and remodeling (Murata et al., 2014; Sun et al., 2015; Waki et al., 2015; Weilner et al., 2015) as well as many various processes, including in cell renewal (Otto et al., 2017; Ran et al., 2017), cell differentiation (Peng et al., 2016; Huang et al., 2017), wound healing (Roy and Sen, 2012), angiogenesis (Pourrajab et al., 2015), or tissue regeneration (Fang et al., 2015). miRNAs are small (16–25 nucleotides) non-coding RNA, often highly conserved among species, that bind to the 3′UTR of target mRNAs controlling to their degradation or translational repression (Huang et al., 2011). Among the several miRNAs reported as regulators of skeleton growth and remodeling, miR-92a was found to be decreased within days after fractures and its *in vivo* inhibition using local or systemic administration of anti-miR-92a in mice with a femoral fracture increased the callus volume, improved neovascularization and overall enhanced the fracture healing (Murata et al., 2014). Fracture healing improvement, with accelerated endochondral ossification, increased callus volume and improved biomechanical parameters, was also reported in a rat model of closed femur fracture with internal fixation, after local injection of rat bone marrow derived mesenchymal stem cells overexpressing miR-21 (Sun et al., 2015). Serum circulating miRNAs (miR-10a-5p, miR-10b-5p, miR-133b, miR-22-3p, miR-328-3p, and let-7g-5p) were also reported with differential levels of expression in patients suffering from osteoporotic fractures; whereas 5 of them were able to regulate the osteogenic differentiation of human mesenchymal stem cells *in vitro* (Weilner et al., 2015). These few examples suggest that miRNAs are key regulators of bone metabolism and may thus display therapeutic interest for bone repair modulation.

### Cell-based therapies

Several approaches, based on cell-therapy, are currently applied to improve both bone healing and bone vessels formation, using multipotent mesenchymal stromal-like cells (MSCs) and/or endothelial progenitor cells (EPCs).

Following the recommendations proposed by the International Society for Cellular Therapy in 2006 (Dominici et al., 2006), cells that exhibit the following properties can be assumed as MSCs: (i) cells that are able to adhere to plastic dishes *in vitro*; (ii) cells that are capable to differentiate into osteoblasts, adipocytes and chondrocytes; and (iii) cells that are CD105^+^, CD73^+^ and CD90^+^ but CD34^−^, CD45^−^, CD14^−^ or CD11b^−^, CD79α^−^ or CD19^−^, and HLA-DR^−^. *In situ*, MSCs contribute to maintenance of the tissue they reside, indicating that while all MSCs exhibit several identical properties, they also have some tissue-specific distinctive characteristics devoted to their residential tissue (Mattioli-Belmonte et al., 2015). They can thus be harvested from several tissues, especially bone and bone marrow, in adults, but also from placenta, umbilical cord blood, adipose tissue, muscle, brain, kidney, heart, and others. Clinical trials are now in the course using MSCs to heal long bones after defects or disunion fractures, or using dental pulp cells in mandible regeneration (d'Aquino et al., 2009; Giuliani et al., 2013; Paino et al., 2017). However, the utilization of MSCs is nevertheless confronted to several complications (Centeno et al., 2010; Yim et al., 2014): (i) their extraction implicates an invasive process and morbidity; (ii) their proliferation and differentiation abilities decrease with the age of the donor; and (iii) lack of early vascularization of MSC associated grafts induces premature death of the implanted cells.

EPCs, mainly found in bone marrow, can mobilize into the bloodstream and home to ischemic sites under the stimulation of specific factors, including G-CSF, VEGF, FGF-2, PGF, EPO, or SDF-1. EPCs are typically described as cells expressing a combination of hematopoietic progenitor markers (CD34/CD133) and an endothelial marker (VEGF receptor-2). These cells are involved in several tissue functions, including neoangiogenesis, vascular repair and blood-flow recovery after ischemia, as well as fracture healing and bone regeneration since they exhibit an osteogenic potential (Atesok et al., 2010; Deng et al., 2011; Keramaris et al., 2012).

Since EPCs represent a very rare population cells in the circulation (0.0001% of the total mononuclear cells in human adult peripheral blood), EPC can be alternatively harvested among the abundant CD34^+^ cell fraction into the adult peripheral blood after G-CSF clinical mobilization. This CD34^+^ cells, also known as endothelial and hematopoietic progenitor cell-rich population, exhibit phenotypic markers associated to both endothelial and hematopoetic lineages (CD133^+^, CD31^+^, c-Kit^+^, CD45^+^, and CD14^−^), and demonstrated angiogenic and osteogenic characteristics similar to EPCs *in vitro* and *in vivo*. Since (neo)vascularization plays a central role in the process of bone development and healing, several studies investigated the ability of CD34+ cells to heal bone fractures and reported encouraging results (Atesok et al., 2010).

### Biomaterials

Several descriptions were proposed for the word “biomaterials.” One definition given by Vert et al. in 2012 is “*material exploited in contact with living tissues, organisms, or microorganisms*” (Vert et al., 2012). Another definition by Williams in 2009 is: “*a biomaterial is a substance that has been engineered to take a form which, alone or as part of a complex system, is used to direct, by control of interactions with components of living systems, the course of any therapeutic or diagnostic procedure, in human or veterinary medicine*” (Williams, 2009). Biomaterials are set to interact with biological structures to assess, increase, restore or heal altered tissues, organs, or functions. Compared to other categories of materials, biomaterials have the properties to persist in their grafted biological situation without harming the surrounding tissues.

A large variety of materials, including natural originated materials, polymers, ceramics, and composites, as well as nano-materials, can be exploited as biomaterials *in vivo* to improve the 3D configuration of the regenerated bone tissue and to promote the cell differentiation along the osteoblastic lineage (Dennis et al., 2015; Khademhosseini and Langer, 2016). The 3D materials need to be biocompatible, porous, and should exhibit the adequate porous architecture to reestablish the global anatomy and function of the original tissues and reduce the production of non-functional necrotic tissues (Hollister, 2005; Kraehenbuehl et al., 2011; Yoo, 2013; Algahtani et al., 2014; Langer, 2015).

Native biomaterials can be a piece of bone from the same individual (autograft), from individuals of the same species (allografts) or from different species (xenografts).

Biopolymers used in tissue engineering are synthetic organic materials biocompatible with human tissues. These materials can be synthetics (such as polylactic acid [PLA], polyglycolic acid [PGA], and copolymers of PLA-PGA), or of natural origin, such as the widely used 3D collagen-based biomimetic hydrogel scaffolds (Helary et al., 2010, 2015). These scaffolds are biocompatible, biodegradable with low antigenicity, and exhibit a suitable environment to provide osteoblast attachment, proliferation, and differentiation (Abou Neel et al., 2013; Mravic et al., 2014). Although collagen scaffolds are *per se* highly hydrated (with more than 95% w/v fluid) with weak mechanical properties for tissue replacement applications (Cheema et al., 2007), the “simple” plastic compression of the material rapidly increases the relative collagen fibrillar density (to more than 10% in weight) by removing the excess of fluid (Brown et al., 2005). The “plastic compression” approach thus yields a type I collagen matrix with a fibrillar density similar to that of native bone matrix (Coyac et al., 2013; Chamieh et al., 2016). This process enables the rapid, controllable and reproducible production of dense collagen gel scaffolds with highly defined meso-structure and increased biomechanical properties, similar to that of the osteoid (Engler et al., 2006). Furthermore, cell seeding constitutes part of the processing route, and the scaffolds provide the 3D structure for their growth and differentiation without compromising their viability (Brown et al., 2005; Ghezzi et al., 2011). At last, it is also important to consider also the use of molecule-entrapped materials that can stimulate or active the tissular resident stem cells. In that purpose, some specific scaffolds, containing growth factors and cytokines, were created to promote the migration and engraftment of host resident stem cells to direct the tissue regeneration (Ko et al., 2013).

Made of non-organic oxides and salts, ceramics are wildly employed in bone tissue engineering due to their resemblance with bone mineral composition in the case of calcium phosphate (a large group of minerals among which hydroxyapatite is the most common) or thanks to their high adhering capacity to bone tissues regarding bioglasses (Miri et al., 2016).

Nanomaterials, including nanoparticles, nanofibers, nanotubes, and nanosheets, have become a popular material to use in tissue engineering (Gaharwar et al., 2014). Nanomaterials are defined as materials with one dimension between 1 and 100 nm. In the bone regeneration field, nanomaterials exhibit several strengths: they are able to mimic the bone nano-composition, they are presenting an increased surface area and roughness, and finally they are displaying strong adsorption properties for cells and bioactive proteins (Zhang and Webster, 2009). Therefore, it has been recently demonstrated that nanoparticles coated at the surface of implant were able to promote osteoblast activity, to decrease osteoclast activity, and finally to enhance bone growth at the interface between the native bone and the implanted device (Alghamdi et al., 2014). Nanomaterials are also presenting osteoinductive properties to promote the osteogenic differentiation of stem cells (Xu et al., 2015), as well as osteoconductive properties to increase the mechanical properties of implantable scaffolds (Wang et al., 2016).

Cells free scaffolds strategies are also developed to promote the ingrowth of new bone. These cell-free scaffolds are designed to provide mechanical stability of implanted material, while promoting osteogenesis, osteoconduction, and/or osteoinduction (Bueno and Glowacki, 2009). These cell-free scaffolds can be “resorbable” and thus be gradually degraded and replaced by new bone (Sandor, 2012; Ros-Tárraga et al., 2016), or “permanent” and thus endure and become integrated within the new bone (Panseri et al., 2013). At last, cell-free materials can incorporate bioactive factors to attract host stem cells, bone ingrowth cytokine to promote osteoformation and/or pro-angiogenesis factors (Patel et al., 2008).

## Angiogenesis and neurogenesis considerations

Since bone tissue is a highly vascularized organ, all the cells that are implicated in vasculogenesis and osteogenesis play a key role in bone formation and remodeling during both prenatal and postnatal times. Therefore, any insufficient blood and cellular supply may delay or impair spontaneous healing of bone fracture. This close relationship, between osteogenesis and angiogenesis, makes neovascularization and ossification-related growth factors important therapeutic mediators for bone healing, including VEGF, FGF-2, BMP-2, -6 and -7 (Wildemann et al., 2007; Cui et al., 2010).

Incorporating or entrapping bioactive factors within biodegradable scaffolds presents the advantage of facilitating their slow release locally over a longer duration (Ko et al., 2013). However, as several growth factors and cytokines are involved, the solely action of only one growth factor could be not sufficient to stimulate the whole bone healing and angiogenesis processes (Geuze et al., 2012). Therefore, the action of several growth factors delivered in the ideal combination could be able to promote both angiogenesis and osteogenesis, thus inducing vessels formation in the tissue constructs (Gorin et al., 2016). It is also imperative to consider cautiously not only the accurate arrangements of these growth factors, but also their administration time, i.e., simultaneous or sequential. At last, it is important to have in mind the use of angiogenic growth factors is also able to stimulate pathological side consequences of angiogenesis promoting tumor development, atheroscleosis, and adverse proliferative pathologies (Moldovan and Moldovan, 2002; Wang et al., 2009; Kilarski et al., 2012).

Instead of promoting angiogenesis using growth factors, one way to favor the vascularization of the grafted construct is to directing add vessel-related progenitors (such as EPCs) (Atesok et al., 2010; Deng et al., 2011; Keramaris et al., 2012; Liu et al., 2017), and/or to artificially create vessels within the biomaterial at the construct step (such as by bioprinting) (Zhu et al., 2017).

Furthermore, it is also important to consider that the grafting environment is poor in oxygen. However, hypoxia is a major stimulus for angiogenesis through the induction of vasodilatation, proliferation, and migration of endothelial cells. Activation of the hypoxia inducible factor (HIF) pathway can trigger transcription of a wide panel of genes, including angiogenic factors such as VEGF and extracellular matrix components (Germain et al., 2010; Gorin et al., 2016).

At last, the restauration of the sensitive innervation of the tissue is important for the future functionality of the implanted engineered tissue (Bataille et al., 2012; Martens et al., 2013, 2014). It has thus be recently reported a calcitonin gene-related peptide (CGRP) positive staining, mainly localized around neovessels, when using DPSC-loaded-dense collagen gel scaffolds after *in vivo* implantation (Gorin et al., 2016).

## Conclusion

The global market of bone graft substitutes is rapidly rising, in particular because of the population demands and the development of the health system. Therefore, one of the most important research and development consideration in tissue engineering is to develop, design and manufacture biodegradable scaffolds.

One of the major challenges would be to improve the scaffold organization in the purpose of fitting with the patient-specific characteristics, as well as to create biocompatible materials with a regular growth rate all along their volume, using pore calibrated gradients or precise dispersions of entrapped cells and/or growth factors (Knothe Tate, 2011). In that purpose, based on their neural crest origin and on their potential to form mineralized tissue, dental pulp stem cells (DPSC) represent an interesting therapeutic tool to restore damaged orofacial bones or teeth (Chamieh et al., 2016). However, many countries start now to request good manufacturing practice (GMP) principles (Giancola et al., 2012) that could greatly increase the complexity of collecting, isolating and incorporating cells into the scaffold, as well as limiting the use of some incorporated growth factors. Finally, the “best” biomaterial scaffold would be pointless without the establishment of a vascular system in the constructs within the following few days after its implantation.

## Author contributions

All authors listed have made a substantial, direct and intellectual contribution to the work, and approved it for publication.

### Conflict of interest statement

The authors declare that the research was conducted in the absence of any commercial or financial relationships that could be construed as a potential conflict of interest. The reviewers VD and VT and handling Editor declared their shared affiliation.
